# Maternal and neonatal characteristics that influence very early neonatal mortality in the Eastern Regional Hospital of Ghana, Koforidua: a retrospective review

**DOI:** 10.1186/s13104-018-3196-x

**Published:** 2018-02-01

**Authors:** James A. Avoka, Richard M. Adanu, Michael Wombeogo, Issah Seidu, Elvis J. Dun-Dery

**Affiliations:** 1 Ghana Health Service, Fanteakwa District Health Directorate, Begoro, Ghana; 2 Department of Population, Family and Reproductive Health, School of Public Health, University of Ghana, Box LG 13, Accra, Ghana; 3 University for Development Studies, Tamale, Ghana; 4 Department of Statistics, University of Ghana, P.O.Box LG 115, Legon-Accra, Ghana

**Keywords:** Very early neonatal mortality, Deaths within 24 h after birth, Early neonatal deaths, Ghana

## Abstract

**Objective:**

This study was conducted to determine the maternal and neonatal characteristics that influence very early neonatal mortality using 811 delivery records at the Eastern Regional Hospital of Ghana.

**Results:**

The very early neonatal mortality rate was 9 per 1000 live births. Multi-parity reduced the odds of very early neonatal death by 30%. Mothers with a previous neonatal death had about 8 times the odds of having a very early neonatal death as compared to those without a history of neonatal death.

## Introduction

Very early neonatal death is any form of newborn mortality that occurs within the first 24 h after delivery [1]. According to World Health Organization (WHO), in developing countries between 25 and 45% of neonates die within the first 24 h after birth [2]. Reports from Ghana clearly indicate that national rate of very early neonatal mortality was 16% per 1000 deliveries [3]. In the Eastern region, very early neonatal mortality rate is 15% per 1000 deliveries [3]. Very early neonatal risk factors such as meconium stained, hypothermia, prematurity, low-birth-weight, infections, asphyxia and birth trauma increased the risk of death in very early neonates [4]. Meanwhile, data to describe the situation within the hospital settings are limited, considering that a comprehensive record review has not been done. This research was conducted to investigate the maternal and neonatal characteristics that influence very early neonatal deaths in the Hospital.

## Main text

### Methods

A record review of neonatal deaths that occurred within the first 24 h after birth was conducted at the Eastern Regional Hospital in Koforidua, Ghana. The study was conducted in this hospital because their annual performance review reports revealed an increasing rate of neonatal mortality at 26, 28 and 35 per 1000 live births for 2012, 2013 and 2014 respectively. Only quantitative data (number of live births, gestational age at booking in weeks, previous neonatal deaths, number of women attending antenatal care (ANC) and postnatal care (PNC), and number of deliveries, birth weight (kg), Apgar score at 1 min and Apgar score at 5 min, etc.) were extracted to obtain and review patients’ records from January, 2013 to December, 2014. All babies delivered and discharged (Dead or alive) within the first 24 h after birth from January, 2013 to December, 2014 were included in the study. However, newborn babies that were referred from other facilities to the Regional Hospital were all excluded. This was because almost all the referred cases did not have complete data required for this study.

All neonatal and maternal characteristics were recorded using data extraction sheets. The data extraction sheet was a manual template developed by the researchers to capture the demographic characteristics such as age of mother, marital status, educational level, religion, occupation, etc. The neonatal characteristics of the babies captured were sex of baby, gestational age in weeks which was determined using the antenatal records. The normal gestational age was determined as (37–42 weeks), and abnormal was (< 37, > 42 weeks), birth weight (kg), Apgar score at 1 min and Apgar score at 5 min. An Apgar score is a rapid general assessment of newborn well-being. It is used instantly after delivery of a newborn baby. These tests are conducted at 1 and 5 min from the time of birth. All deaths occurring within the first 24 h were marked as variables of interest. Data analyses were conducted using STATA SE 13. Measures of association were made using Chi square test. Bivariate associations between Apgar scores and all other variables under study were determined using a significance level of P < 0.05 and 95% confidence interval. Odds ratios and confidence intervals were computed to determine the strength of association between the risks of death and survival. The very early neonatal mortality rate was calculated using the standard formula [1, 5]:

$${\text{VENMR}} = \frac{\text{Total number of VENMs}}{\text{Total live births}} \times 1000.$$

#### The review process

The researchers sampled all records of live birth, giving 9961 live births for the period under review. The researchers further selected the total number of babies referred to the neonatal intensive care unit but excluded all folders with missing cases and those with wrong entry (this was determined with the support of facility staff), giving a total 811 neonates. Folders were further selected based on neonates who survived and those who died within the first 24 h after birth. This resulted in a total of 87 very early neonatal deaths.

### Results

#### Demographic characteristics

The demographic characteristics show that majority of women who went through delivery fell between the ages of 18–35 years making up 77.4% of pregnant women. The teenagers formed 10% of the deliveries and those above 35 years formed 12.6%. Majority of the women had Secondary/Technical/College certificates making up 38.6% of the records reviewed. 92.6% of the women who delivered at the Hospital were Christians and employment rate was 72.7%. A little over twenty (20.3%) percent of the women have ever had their children dying at infancy. Table 1 below gives a detail account of the results (See Table 1).

**Table 1:** Demographic characteristics of postpartum women from records review

	Frequency	Percent (%)
Maternal age		
Below 18	81	10.0
18–35	628	77.4
Above 35	102	12.6
Education level		
No formal	227	28.0
Primary	245	30.2
Secondary/Technical/College	313	38.6
University	26	3.2
Religion		
Christian	751	92.6
Muslim	57	7.0
Traditional	3	0.4
Job status		
Employed	590	72.7
Unemployed	221	27.3
Number of previous child deaths		
None	646	79.7
One or more	165	20.3
Marital status		
Married	570	70.3
Not married	241	29.7

#### Maternal characteristics and neonatal deaths

Out of the total number of 811 records reviewed, 89% of the neonates survived and 11% died within the first 24 h after delivery. Male children formed the majority (56.4%) of the deaths. At 1 min, among those with normal Apgar scores, 12.1% of neonates were alive while 2.3% died. Six hundred and seventeen (n = 617) of those with low Apgar score survived (87.9%). A series of conditions such as pre-term (30.9%), low Apgar score of 24.3%, and neonatal Asphyxia (20.2%), accounted for neonatal intensive care unit (NICU) admissions (Table 2).

**Table 2:** Bivariate analysis showing maternal and neonatal characteristics of mothers who lost their babies as compared to those who survived within 24 h after birth

Attribute	Mortality status				Chi square	P value
	Survived		Died			
	Freq.	%	Freq.	%		
Antenatal status						
Non-attendant	36	5.0	8	9.2	2.652	0.266
Attendant	640	91.3	76	87.4		
Not regular attendant	26	3.7	3	3.4		
Gestational age (weeks)						
Normal	470	67.0	46	52.9	6.780	0.009
Preterm	232	33.0	41	47.1		
Previous neonatal death						
No	660	94.2	77	88.5	4.075	0.044
Yes	42	5.8	10	11.5		
Birth weight						
Normal (≥ 2.5 kg)	366	52.1	32	36.8	7.031	0.007
Low (< 2.5 kg)	336	47.9	55	63.2		
Apgar score 1 min						
Normal (≥ 6)	85	12.1	2	2.3	7.592	0.006
Low (< 6)	617	87.9	85	97.7		
Apgar score 5 min						
Normal (≥ 6)	204	29.1	7	8.0	17.448	0.000
Low (< 6)	498	70.9	80	92.0		

#### Bivariate analysis showing maternal and neonatal characteristics of mothers who lost their babies as compared to those who survived within 24 h after birth

For maternal characteristics, the age of the mother showed a significant association with very early neonatal mortality (P = 0.032). Those below 18 years were associated with fewer deaths than those above 18 years (P = 0.032). Again, parity was significantly associated with very early neonatal mortality (P = 0.002). Additionally, very early neonatal death was also associated with low birth weight (P = 0.007). Furthermore, a woman’s experience of previous child death significantly determined the mortality of her next child within the first 24 h after birth (P = 0.001). An antenatal status was meant to verify whether the child’s mother was attending antenatal care (ANC) before delivery. If she was, then we went further to investigate whether she was a regular attendant (4 ANC visits) or not (less than 4 ANC visits). Also, gestational age was determined in weeks using the antenatal records. The Normal gestational age was determined as (37–42 weeks), and abnormal was (< 37, > 42 weeks). The Apgar score at 1 min ranges from normal (≥ 6) to low (< 6) and Apgar score at 5 min normal (≥ 6) and low (< 6) (see Table 2).

#### Maternal and neonatal characteristics that influence very early neonatal death (multiple logistic regressions)

Multi-parity (more than one delivery) reduced the odds of very early neonatal death by 30% (P < 0.02, OR 0.69, 95% CI 0.52–0.9). However, mothers of lower educational status were less likely to experience very early neonatal death (P < 0.30, OR 1.07, 95% CI 0.88–1.64), but those with previous neonatal death (P < 0.001, OR 7.93, 95% CI 2.83–22.19) were significantly associated with very early neonatal deaths. However, gestational age (P < 0.69, OR 0.86, 95% CI 0.41–1.78) and antenatal clinic attendance status (P < 0.12, OR 0.66, 95% CI 0.39–1.11) were not significant factors determining very early neonatal deaths. The very early neonates with low birth weights were 2.1 times the odds of dying within the first 24 h after birth (P < 0.04, OR 2.07, 95% CI 1.21–4.34). Neonates with low 1 min Apgar score were 69% times the odds of experiencing very early neonatal death (P < 0.03, OR 1.69, 95% CI 0.65–3.56) and 5 min Apgar score had about 7 times the odds of experiencing very early neonatal death (P < 0.002, OR 6.67, 95% CI 3.78–10.43) (see Table 3).

**Table 3:** Logistic regression showing significant risk factors that influence very early neonatal mortality

Factors	Coefficient	Odds ratio	95% CI		P value
Maternal age	− 0.009	0.99	0.94	1.05	0.769
Parity	− 0.358	0.70	0.52	0.94	0.020
Number of child deaths	3.548	34.75	18.22	66.24	< 0.001
Education level	0.216	1.07	0.88	1.64	0.301
Antenatal status	− 0.423	0.66	0.39	1.11	0.117
Gestational age	− 0.151	0.86	0.41	1.78	0.685
Previous neonatal death	2.07	7.93	2.83	22.19	< 0.001
Marital status	0.37	1.45	0.71	2.94	0.304
Job status	− 0.162	0.85	0.44	1.65	0.631
Birth weight	0.728	2.07	1.21	4.34	0.041
Apgar 1 min status	0.332	1.39	0.65	3.56	0.033
Apgar 5 min STATUS	1.898	6.67	3.78	10.43	0.002
Constant	− 4.065	0.017			< 0.001

## Discussion

This study sought to unveil the maternal and neonatal characteristics influencing deaths that occur within 24 h after birth. The study revealed that out of the total number delivered, over nine thousand (n = 9656) of them were live births (97%). This agrees with a study [6] that shows that out of 5845 newborns that were delivered; 5689 of them were live births (97%). This generally implies that deliveries conducted by skilled attendants give very high positive outcomes.

The very early neonatal mortality rate was therefore found to be 9 per 1000 live births as against the National and Eastern Regional figures of 16% per 1000 live births (160 out of 1000 live births) and 15% per 1000 live births (150 out of 1000 live births) respectively [3]. This implies that the hospital is doing better to ameliorate very early neonatal survival. The results of the study also agrees with the findings of the Afghan study [5] which indicates a very early neonatal mortality rate of 7.4 per 1000 live births which is a little lower than findings of this study. The very early neonatal death rate is rather high if compared with the United States of America (USA) which is 0.91 and 1.58 per 1000 live births [7].

Multi-parity reduced the odds of very early neonatal death by 30% as compared to the primiparas. These results support the findings [4] which revealed that parity is associated with very early neonatal mortality. Gestational age and mothers who have had previous neonatal deaths were significantly associated with very early neonatal deaths. The study is consistent with the findings [8] by revealing that number of previous child and neonatal deaths were associated with very early neonatal mortality.

Apgar score at 5 min was the main consequence of risk factors contributing to very early neonatal mortality. Other studies have tried to establish an association between multi-parity and low Apgar score at 5 min [9]. Earlier studies have investigated the factors influencing associations between 1 min Apgar score and poor neonatal outcome and have identified birth weight, and prematurity to be the neonatal and maternal factors contributing to deaths among neonates which is partially consistent with this current study [10]. Low birth weight babies had 2.07 times the odds of death as compared with normal weight babies which is consistent with the study conducted in Nigeria and Brazil [8, 11].

### Conclusion

In conclusion, births within the first 24 h are a critical stage to earmark for interventions and therefore, the babies need to be critically monitored and supported within the first 1 h after birth. The women who gave birth more than once (Multiparas) were less likely to have their very early neonates dying within 24 h after birth as compared to the primiparas (first time deliveries). This may indicate a lack of experience in timely breastfeeding initiation among the primiparas leading to the very early neonatal deaths in the first 24 h of life after birth. Mothers who have ever had any of their children dying at infancy were more likely to experience very early neonatal mortality as compared to women who have never recorded any. Further research is needed to unravel the factors behind it.

### Limitations

The study failed to assess the hospital data on still births, which could have influenced the prevalence of very early neonatal deaths. Mothers of the children were not also interviewed; therefore their opinions about very early neonatal deaths were not recorded as part of the study results. Again, the study was only limited to very early neonatal mortality and did not assess mortality among newborns who survived after 24 h of birth. Additionally, data collection was limited to a single hospital and therefore generalization of the findings is limited but could be considered indicative of the context considered.

## Abbreviations

WHO: World Health Organization
ANC: antenatal care
PNC: postnatal care
VENMR: very early neonatal mortality rate
NICU: neonatal intensive care unit
CI: confidence interval
OR: odds ratio
USA: United States of America

## References

[1] WHO. Intrapartum and very early neonatal death rate. MEASURE evaluation PRH/Family planning and reproductive health indicators database. 2010. http://www.cpc.unc.edu/measure/prh/rh_indicators/specific/nb/intrapartum-and-very-early-neonatal-death-rate. Accessed 13 Aug 2014.

[2] WHO. Newborns: reducing mortality. Fact sheet. 2016. Retrieved from WHO Media centre, January 2016.

[3] Ghana Health Service (2014). Annual performance review report, 2014.

[4] Mosha TCE, Philemon N (2010). Factors influencing pregnancy outcomes in Morogoro Municipality, Tanzania. Tanzan J Health Res.

[5] Kim YM, Ansari N, Kols A, Tappis H, Currie S, Zainullah P, Bailey P, Semba R, Sun K, van Roosmalen J, Stekelenburg J (2013). Assessing the capacity for newborn resuscitation and factors associated with providers’ knowledge and skills: a cross-sectional study in Afghanistan. BMC Pediatr.

[6] Langli H, Mduma E, Svensen E, Perlman JM (2012). Early initiation of basic resuscitation interventions including face mask ventilation may reduce birth asphyxia related mortality in low-income countries: a prospective descriptive observational study. Resuscitation.

[7] Oza S, Cousens SN, Lawn JE (2013). Estimation of daily risk of neonatal death, including the day of birth, in 186 countries in 2013: a vital-registration and modeling-based study. Lancet Glob Health.

[8] Kassar SB, Melo AMC, Coutinho SB, Lima MC, Lira PIC (2013). Determinants of neonatal death with emphasis on health care during pregnancy, childbirth and reproductive history. J Pediatr.

[9] Salustiano EMA, Campos JADB, Ibidi SM, Ruano R, Zugaib M (2012). Low Apgar scores at 5 minutes in a low risk population: maternal and obstetrical factors and postnatal outcome. Artigo Orig.

[10] Jeganathan R, Karalasingam SD, Hussein J, Allotey P, Reidpath DD (2017). Factors associated with recovery from 1 minute Apgar score < 4 in live, singleton, term births: an analysis of Malaysian National Obstetrics Registry data 2010–2012. BMC Pregnancy Childbirth.

[11] Adetola AO, Tongo OO, Orimadegun AE, Osinusi K (2011). Neonatal mortality in an urban population in Ibadan Nigeria. Pediatr Neonatol.

